# Serum autoantibodies against α7-nicotinic receptors in subgroups of patients with bipolar disorder or schizophrenia: clinical features and link with peripheral inflammation

**DOI:** 10.1038/s41398-024-02853-8

**Published:** 2024-03-14

**Authors:** Estelle Darrau, Elise Jacquemet, Stéphanie Pons, Laurène Schlick, Marios Zouridakis, Ching-Lien Wu, Jean-Romain Richard, Caroline Barau, Philippe Le Corvoisier, Robert Yolken, Ryad Tamouza, Marion Leboyer, Uwe Maskos

**Affiliations:** 1 Université Paris Est Créteil, INSERM U955, IMRB, Translational NeuroPsychiatry Laboratory, Créteil, France; 2 Institut Pasteur, Université de Paris Cité, Integrative Neurobiology of Cholinergic Systems, CNRS UMR 3571, Paris, France; 3 Institut Pasteur, Université de Paris Cité, Bioinformatics and Biostatistics Hub, Paris, France; 4 Laboratory of Molecular Neurobiology and Immunology, Hellenic Pasteur Institute, Athens, Greece; 5 AP-HP, Hôpitaux Universitaires Henri Mondor, Département Médico-Universitaire de Psychiatrie et d’Addictologie (DMU IMPACT), Fédération Hospitalo-Universitaire de Médecine de Précision en Psychiatrie (FHU ADAPT), Creteil, France; 6 Fondation FondaMental, Creteil, France; 7 Plateforme de ressources biologiques, Hôpital Henri Mondor, Université Paris Est Créteil, Creteil, France; 8 Centre d’Investigation Clinique 1430, AP-HP, Hôpital Henri Mondor, Université Paris Est Créteil, Créteil, France; 9 Stanley Division of Developmental Neurovirology, Department of Pediatrics, Johns Hopkins School of Medicine, Baltimore, MD USA

**Keywords:** Physiology, Molecular neuroscience

## Abstract

There is growing evidence that autoantibodies (AAbs) against proteins expressed in the brain are playing an important role in neurological and psychiatric disorders. Here, we explore the presence and the role of peripheral AAbs to the α7-nicotinic acetylcholine receptor (nAChR) in inflammatory subgroups of psychiatric patients with bipolar disorder (BD) or schizophrenia (SCZ) and healthy controls. We have identified a continuum of AAb levels in serum when employing a novel ELISA technique, with a significant elevation in patients compared to controls. Using unsupervised two-step clustering to stratify all the subjects according to their immuno-inflammatory background, we delineate one subgroup consisting solely of psychiatric patients with severe symptoms, high inflammatory profile, and significantly increased levels of anti-nAChR AAbs. In this context, we have used monoclonal mouse anti-human α7-nAChR antibodies (α7-nAChR-mAbs) and shown that TNF-α release was enhanced upon LPS stimulation in macrophages pre-incubated with α7-nAChR-mAbs compared to the use of an isotype control. These findings provide a basis for further study of circulating nicotinic AAbs, and the inflammatory profile observed in patients with major mood and psychotic disorders.

## Introduction

Mental disorders are the most debilitating and costly diseases in developed countries as they affect around 40% of the European population at some point in their lives [1]. Among major mood and psychotic disorders, SCZ and BD are complex to manage because of their clinical heterogeneity and the absence of any valid biomarkers to choose the most adapted treatments [2]. So far, these complex disorders remain diagnosed only upon clinical criteria and are mainly treated with pharmacological agents. Nowadays, pharmacological treatments used to treat SCZ and BD are relying on a few classes of drugs and are not effective in all patients. Indeed, some studies showed that one-third of patients with SCZ and one-quarter with BD are treatment-resistant [3–5].

Consequently, the identification of homogeneous subgroups and our understanding of the causes of psychotic disorders remain limited. Hence, the development of new pathway-related treatments is hampered. Since the last decade, growing evidence shows that immune system dysfunctions are directly associated with psychotic disorders. Genetic studies highlight a strong association between SCZ and the Major Histocompatibility Complex (MHC) far ahead of any genes expressed in the central nervous system (CNS) [6]. Growing evidence suggests that pro-inflammatory mediators such as tumor necrosis factor (TNF) and interleukins (IL), such as IL-1β, IL- 6, or IL-8, could play a role in the pathophysiology since these mediators are increased in SCZ and BD compared to healthy subjects [7, 8]. These findings open up new avenues for the discovery of biomarkers, the understanding of mechanisms and the implementation of innovative treatments [9, 10]. These links have been reinforced by the co-occurrence of inflammation and autoimmune disorders with psychotic disorders [3, 11, 12], and in particular by the discovery of anti-neuronal AAbs such as the anti-N-methyl d-aspartate receptor autoantibodies (NMDA-R-AAbs) that cause severe neurological or psychiatric disorders, respectively referred to as autoimmune encephalitis [13] or autoimmune psychosis [14, 15]. These findings led to the discovery of other AAbs against CNS proteins associated to neurological and psychiatric manifestations [16, 17]. In this rapidly evolving field, we wanted to provide new evidence that AAbs targeting nAChRs are associated with major mood or psychotic disorders.

The nAChRs are pentameric ligand-gated ion channels widely expressed throughout the human body that are at the origin of one of the first established autoimmune diseases called “*myasthenia gravis”* involving AAbs against muscle nAChR [18]. This immune response is most likely due to a very immunogenic fold in the extracellular domain of the nAChR, termed the *main immunogenic region* [19]. In 2000, the first AAbs against *neuronal* nAChRs were described targeting the peripheral ganglia, and leading to autoimmune autonomic neuropathies [20]. Today there is an unmet need to better characterize the role of anti-nAChR AAbs. Humans exhibit numerous neuronal pentameric nAChRs, composed of different combinations of α2-α7, α9-α10, and β2-β4 widely expressed in the CNS, but also found in epithelial tissues, peripheral immune cells such as monocytes/macrophages, lymphocytes, dendritic cells, and brain-resident microglia [21]. They are stimulated by acetylcholine (ACh) released by lymphoid cells and the vagus nerve [22]. nAChRs appear to play a role in the generation of antibodies with a possible inhibitory regulatory role of the α7 subtype [23]. Moreover, the stimulation of this α7-nAChR subtype expressed at the surface of macrophages via the electric stimulation of the vagus nerve inhibits the release of pro-inflammatory cytokines (TNF-α, IL-1-b, IL-10, or IL-8) from macrophages as part of the so-called cholinergic anti-inflammatory pathway [24]. Murine models comparing α7-nAChR knock-out mice to wild-type showed that the stimulation of the vagus nerve did not alleviate the secretion of lipopolysaccharide (LPS)-triggered TNF-α in knock-out mice [25]. Furthermore, the anti-inflammatory effect of a direct application of nicotine was abolished in the monocyte-derived macrophages from α7-nAChR knock-out mice stimulated in vitro with LPS. In addition, α7-nAChR knock-out mice exhibited abnormal immune responses including metabolic disturbances, increased production of IgG1 and pro-inflammatory cytokines [23, 26].

A comparable regulatory pathway has been described for in vitro culture of brain mononuclear phagocytic cells, the microglia, as stimulation of α7-nAChR with direct application of ACh suppresses LPS-induced TNF-α release [27]. In this context, vagus nerve stimulation has been used to alleviate inflammation in severe immune-inflammatory disorders but also to improve depressive symptoms in patients with severe treatment-resistant depression [24].

In humans, there is a unique partially duplicated gene that includes the fusion between the α7-nAChR (exon 5-10) and the FAM7A (exons A-E) called *CHRFAM7A* coding for the truncated *dupα7* protein [28]. The CHRFAM7A/CHRNA7 ratio is increased in human macrophages stimulated with LPS and in subjects suffering from certain chronic inflammatory diseases, such as inflammatory bowel diseases. Interestingly this ratio seems to be also increased in patients with SCZ and BD compared to controls, however, there is no details about their inflammatory profile [29–31].

Taken together, these studies raise the question of possible α7-nAChR damage in SCZ or BD patients, which could impact both the brain and the immune system and thus promote the onset of psychiatric and immuno-inflammatory symptoms. Previous studies performed in 21 SCZ patients, 26 patients with Alzheimer disease, 19 children with obstructive bronchitis and two patients with Rasmussen encephalitis provided evidence for the first time that AAbs targeting central neuronal α7-nAChR AAbs can be detected in serum by using enzyme-linked immunosorbent assay (ELISA) [32–34]. Similarly to the functional effects of NMDA-R AAbs in autoimmune encephalitis, these AAbs would potentially block the function of the corresponding nAChR. In animal studies, α7-nAChR knock-out mice exhibited abnormal immune responses, including metabolic disturbances, increased production of IgG1 and pro-inflammatory cytokines [23, 26].

This initial evidence prompted us to explore whether α7-nAChR AAbs are associated with a specific immuno-inflammatory profile in a subgroup of psychiatric patients with low-grade inflammation. To address these questions, we have measured serum α7-nAChR AAbs, anti-neurotropic and gut pathogen signatures as well as a large array of cytokines/chemokines along with assessments of clinical symptoms in a cohort of hospitalized and ambulatory SCZ and BD patients, as well as healthy controls (HC). First, we have used an unsupervised two-step clustering to stratify all the subjects according to their immuno-inflammatory background into homogeneous subgroups to dissect their clinical and serological phenotype without specifying the diagnosis and the status of hospitalization for patients. As an experimental validation, we have further examined whether mAbs targeting human α7-nAChR (i) could enhance the production of cytokines by macrophages stimulated with LPS from *E. coli* and (ii) could abolish the anti-inflammatory effect of nicotinic agents currently used to stimulate the α7-nAChR by preventing its binding to its cognate receptor.

## Materials and methods

### Population

A total of 584 subjects were enrolled in the present study, including 149 healthy controls (Centre Investigation Clinique, Henri Mondor Hospital, Créteil) and 435 psychiatric patients. Of these, 166 (28%) were included during a standardized assessment in an Experts Center coordinated by Fondation Fondamental, and 269 (46%) during a hospitalization (Psychiatry Department, Henri Mondor University Hospital, Créteil, France). The inclusion criteria were as follows: age between 18 and 65 years, no pregnancy or breastfeeding, no vaccination in the 4 weeks before the participation, no ongoing immunomodulatory or immunosuppressive treatment, no concomitant neurological/neurodegenerative disorder, and furthermore, for the healthy controls, no ongoing autoimmune or psychiatric disorder, including alcohol and drug addiction. For the psychiatric patients, only subjects with a current diagnosis of schizoaffective disorder, bipolar disorder or schizophrenia according to DSM-IV-TR criteria (American Psychiatric Association) were included in the present study. Blood samples were collected the day of the inclusion and sent to the “Centre de ressources biologiques”, Henri Mondor Hospital, Créteil, France. The sera were kept at −80 °C before being sent to the different partner laboratories. The French cohort I-GIVE (Immuno-Genetics, Inflammation, retro-Virus, Environment; n°EUDRACT 2013-A01639-36, Fondation Fondamental, Créteil) was approved by the French local Ethics Committee (n°Am7157-3-3139, Comité de Protection des Personnes Ile-de-France III, Hôpital Tarnier-Cochin, Paris) and written informed consent was obtained from each study participant.

### Sociodemographic and clinical assessments

The day of inclusion, after the informed consent was signed and the blood sample was collected, the subject was interviewed by the investigator. Concerning patients, the manic symptoms were measured with the Young–Mania Rating Scale (YMRS) and depressive symptoms with the Montgomery–Åsberg Depression Rating Scale (MADRS). Psychotic symptoms were assessed with the Positive and negative syndrome scale (PANSS), and symptom severity was assessed by using the Functional assessment staging tool (FAST), the Global assessment functioning (GAF symptom and GAF handicap scales), and the clinical global impression (CGI). Concerning healthy controls, schizotypal personality was assessed with the auto-questionnaire “Schizotypal personality disorder questionnaire” (SPQ).

### Circulating inflammatory markers and infectious stigmas

The cytokines (GM-CSF, IFN-γ, TNF-α, TNF-β, IL-1α, IL-1β, IL-2, IL-4, IL-6, IL-7, IL-8, IL-10, IL12/23p40, IL-12p70, IL-13, IL-15, IL-16, VEGF, and IL-17), B cell-activating factor (BAFF) and adhesion molecules encompassing soluble intercellular adhesion molecule 1 (sICAM1)/soluble vascular cell adhesion molecule 1 (sVCAM1) were measured by electrochemiluminescence assay (MSD, Rockville, MD, USA). Each sample was run in duplicates, and all the plates were read with the MSD workbench and the software package recommended by the manufacturer. Beta-2-microglobulin was measured by standard Enzyme-linked-immunosorbent assay (ELISA) (BioVendor, Brno, Czech Republic). The qualitative detection of previous infections with neurotropic pathogens were measured with ELISA. Antibody titers (immunoglobulin G/IgG) against Herpes simplex 1 (HSV-1), cytomegalovirus (CMV) and Toxoplasma gondii (Toxo) were calculated according to the cutoff given by the kit [35]. The occurrence of anti-saccharomyces antibodies was assessed by qualitative ELISA (Aesku Lab, Wendelsheim, Germany).

### Serum anti-nAChR AAb measurements

The anti-α7-nAChR-AAs were measured by an ELISA developed for this study. Briefly, 25 µg of the deglycosylated pentameric extracellular domain of the α7-nAChR [36] in PBS 1× was incubated overnight at 37 °C in 96-well plates (Thermofisher, Waltham, MA, USA). The plates were washed with PBS-Tween 0.1% and blocked with 5% BSA (Sigma-Aldrich, MO, USA) in PBS 1× and washed twice. Human serum and control antibodies were added in duplicates and incubated 2 h at 37 °C.

The samples of human serum include a wide variety of Abs directed against different epitopes of the α7-nAChR with different affinities. Consequently, we chose to not perform a quantitative ELISA test with a reference standard curve but opted to introduce an identical and quantifiable positive control in all the tests carried out. This positive control allows us to validate each of the tests and to carry out a standardization to render each human sample comparable. This positive control is also used to validate the test itself and to quantify its reproducibility. The positive controls were polyclonal rabbit anti-human-α7-nAChR antibody (Ab10096, Abcam Cambridge, UK) and a monoclonal mouse anti-human-α7-nAChR (ma531691, Thermofisher, Waltham, MA, USA), used at 1 and 0.1 µg/ml. Taking the monoclonal positive control as a measure, the detection limit is better than 0.35 nM. The average value of the OD of the positive control for all the tests carried out is 0.578 with a SD of 0.110 and a SEM of 0.02.

After washes, the plates were incubated with secondary polyclonal antibodies (Peroxidase AffiniPure Goat Anti-Human IgG, F(ab’)2 fragment specific (cat#109005006, JacksonImmunoResearch, Cambridgeshire, UK), or HRP-Goat anti-Rabbit or HRP-Goat anti-mouse IgG (H + L) secondary antibody (Cat# 31460 & Cat# 31430, Thermofisher, Waltham, MA, USA). The revelation was made with OPD-buffer and stopped with H2SO4 (Sigma-Aldrich, MO, USA). The optical density (OD) was read at 490 nm.

### Data treatment and clustering analyses

For the clustering analyses, cytokines and AAb concentrations were normalized with a Box–Cox transformation [37] to limit the impact of data skewness (Supplementary Fig. 1). To cluster the individuals on their inflammatory profile, we first performed a principal component analysis (PCA) with all the observations for the 20 cytokines and the anti-α7-nAChR-AAbs to group cytokines into dimensions and reduce variability. We then generated a hierarchical ascendant classification (HAC) on the five first dimensions of the PCA using Euclidian distance and ward D criterion to identify inflammation-based clusters. The partitioning was then consolidated using a k-means algorithm to further ensure the homogeneity between patients within each cluster. To do so, the algorithm was run using the average individual for each initial cluster as starting point. We finally used general descriptive statistics and visualizations to describe and interpret the emerging clusters regarding clinical data available. We kept the first five dimensions which corresponded to 53.5% of explained variance in the model (Fig. 3a–c). All statistical analyses and visual representations were conducted using the R Statistical language (version 4.0.5; R Core Team, 2021) on Ubuntu 20.04.5 LTS. For in vitro experiments, data were analyzed with GraphPad Prism (version 8.0.0, La Jolla, CA) for Windows, either one-way ANOVA with Holm–Sidak post hoc test or Kruskal–Wallis with Dunn’s test for multiple comparisons were used with statistical significance set at *P* < 0.05.

### Cell culture and antibodies

We used the THP-1 monocyte-like cell line (TIB-202, ATCC) derived in macrophages for in vitro experiments. Cells were cultured in T75 cell-culture flasks in a humidified atmosphere of 5% CO_2_ at 37 °C and passaged every week. THP-1 were grown in complete RPMI 1640 medium (Gibco, Grand Island, NY) supplemented with 10% heat-inactivated fetal bovine serum, 20 mM l-glutamine (Gibco, Grand Island, NY), β-mercaptoethanol and 5% penicillin/streptomycin (Gibco, Grand Island, NY) according to the recommendations from ATCC. For experiments, cells were seeded at 7 × 10^5^ cells/ml in 96-well plates and differentiated into macrophage-like cells with 100 nM phorbol 12-myristate 13-acetate (PMA, Tocris Bio-techne, MN, USA) in complete medium for 24 h and in PMA-free complete medium for 48 h. The release of TNF-α was stimulated by using 1 µg/ml of LPS E. coli (O111, B4, Sigma-Aldrich, MO, USA). TNF-α secretion was measured by ELISA (TNF-alpha DuoSet ELISA, R&D Systems, MN, USA) according to the manufacturer’s instructions. In order to select a nicotinic drug with the most efficient anti-inflammatory effect, we pre-incubated macrophages with increasing concentrations of the following pharmacological components: PNU-282987, PNU-120596, and NS6740 (supplied by Tocris Bio-techne, MN, USA).

In the experimental conditions, the macrophages were pre-incubated with 10 µg/ml of mouse monoclonal IgG1 anti-human α7-nAChR (α7-nAChR mAb/MA531691) or its isotype control (IC-mAb, Rockland Immunochemicals, PA, USA) for 2 h before LPS incubation and LPS + NS6740 incubation.

### Immunostaining

**Fig. 1:**
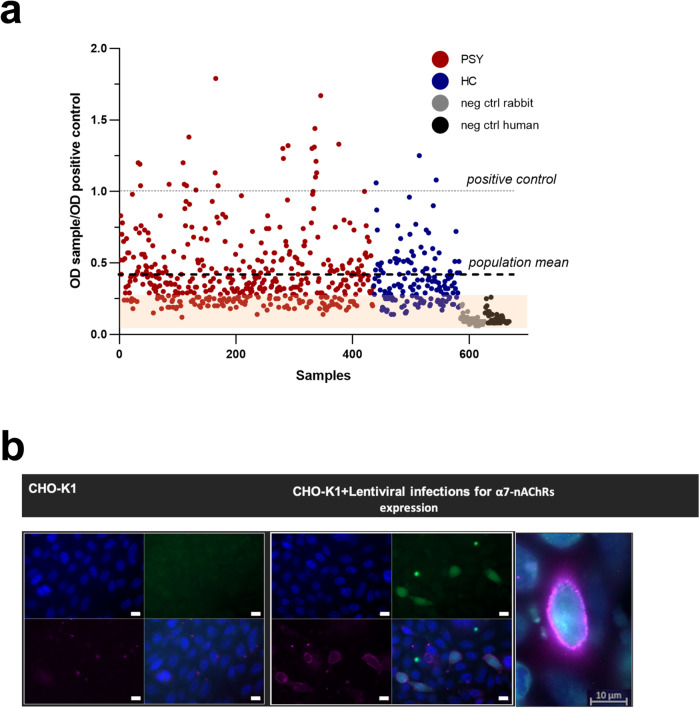
Anti-α7-nAChR IgGs in blood samples. **a** Serological reactivity of human serum on the α7-nAChR extracellular domain measured by ELISA, the values equal to the background noise are indicated by the orange square (PSY psychiatric patients, HC healthy controls, neg ctrl rabbit goat anti-rabbit secondary antibody with HRP, neg ctrl human = goat anti-human secondary antibody HRP). Sample ODs ranged from 0.12 to 1.79. **b** Immunostaining of 1 µg/ml of monoclonal anti-human α7-nAChR on CHO-K1 cells expressing or not the human α7-nAChR labeled with secondary goat anti-mouse IgG-AlexaFluor 647 (scale bar = 10 µm).

The specificity of the α7-nAChR-mAb was determined by immunostaining on CHO-K1 cells (ATCC – CCL-61; Manassas, USA) cultured with DMEM/F12 supplemented with 10% FBS and antibiotics (Gibco, Grand Island, NY) modified by lentiviral infections to be able to constitutively express high levels of human α7-nAChR at the plasma membrane (Fig. 1a). The CHO cells cultured on glass coverslips coated with poly-l-lysine and collagen were washed with OptiMEM (Gibco, Grand Island, NY) and incubated 3 h with 100 nM of α-bungarotoxin-alexa fluor 647 (Invitrogen, Life Technologies, OR, USA) or 1 µg/ml of monoclonal mouse anti-human-α7-nAChR Ab at 37 °C. After three washes in PBS 1×, the cells were fixed with 2% PFA 15 min at room temperature and mounted onto microscope slides with ProLong gold with DAPI (Invitrogen, Life Technologies, OR, USA). For the condition with α7-nAChR Ab, the cells were saturated 1 h at room temperature with a blocking PBS 1× buffer containing 1% NHS (Gibco, Grand Island, NY) and 0.2% gelatine (Sigma-Aldrich, MO, USA) and incubated 2 h with secondary goat anti-mouse-AlexaFluor-647 (JacksonImmunoResearch, Cambridgeshire, UK) in blocking buffer before being mounted as previously described. Cells were imaged with an inverted fluorescent microscope with the far-red channel using the software Zen Axiovision (Zeiss, Léna, Germany).

## Results

### Population

Sociodemographic characteristics are listed in Table 1. The studied population was composed of patients with BD (*N* = 286, 49%) and an equal proportion of SCZ (*N* = 149, 25.5%) and HC (*N* = 149, 25.5%). Overall, almost half of the patient population has been recruited during a hospitalization for an acute episode. Patients were on average older than HC (40.2 ± 13.5 vs 35.3 ± 13.1, *W* = 39,170, *P* value = 0.0001), and both populations were similar regarding sex ratio (Table 1).

**Table 1:** Characteristics of the study sample.

Study sample		
	435 Psychiatric patients	149 Healthy controls
*N*	435 (74%)	149 (26%)
Age^a^	40.24 (13.5)	35.3 (13.1)***
Sex^b^	44.1%	51.0%
Tobacco users	29.2%	14.8%
Clinical features		
Age of onset^a^	28.55 (10.98)	
Illness duration^a^	15.52 (12.52)	
SZ	149 (26%)	
BD	286 (49%)	
HC	149 (26%)	
Inclusion	*N*	
Hospitalization	269 (46%)	
Consultation	166 (28%)	
CIC	149 (26%)	
Treatments^c^		
% Anticholinergics	17/366 (4.6%)	
% Clozapine	9/366 (2.5%)	
% Chlorpomazine	5/366 (1.4%)	
Serum IgGs		
Anti-α7-nAChRs AAs^a,d^	0.44 (0.3)*	0.38 (0.2)
Cutoff^e^	45/435 (10.34%)*	6/149 (4.03%)
Cutoff SZ	12/149 (8.05%)	
Cutoff BD	33/286 (11.53%)	
Infectious stigmas		
Anti HSV Pos%	58.1%	62.4%
Anti-CMV Pos%	58.9%	64.5%
Anti-Toxo Pos %	64.3%	54.2%
Anti-ASCA Pos %	20.5%	11.4%

***P < 0.0001; *P < 0.05.

^a^Mean (sd).

^b^% female.

^c^%.

^d^Kruskal-Wallis; 4.1187; df 1; *p* = 0.04241.

^e^Pearson’s Chi-Squared; 4.7939; df 1; *p* = 0.02856.

### Relationship between circulating AAbs targeting the pentameric preparation of α7-nAChR extracellular domain, cytokines, and diagnoses

Overall, 65% of all the serum samples tested presented an OD superior to the mean of the OD of negative controls, while 35% of the samples were in the plate background (orange square) according to the OD of the two negative controls (Fig. 1a). Despite the scattered distribution, the psychiatric patients have on average significantly higher OD values compared to HC (0.44 ± 0.3 vs 0.38 ± 0.2, Wilcoxon rank-sum test with continuity correction *W* = 28,800, *P* value = 0.04244; Fig. 1a and Table 1). In order to limit inter-plate variability, we normalized the OD obtained for each subject with the OD of the positive control (1 µg/ml). The specificity of the anti-α7-nAChR Ab was validated both in ELISA and immunostaining to verify its binding to the native receptor (Fig. 1b). Based on the paper of Chandley and colleagues [32], we used an arbitrary cutoff calculated as follows: mean of HC + 2 standard deviations to highlight the subjects with values higher than the average presented by the healthy population. The results showed that a higher percentage of patients with psychiatric disorders was above the cutoff compared to HC; however, the proportions were similar between BD and SZ (Table 1). In order to study the relationship between the α7-nAChR AAbs OD, the concentration of cytokines and the diagnosis by controlling confounding variables, we used a multiple linear regression and found that three cytokines (IL-4, IL-7, IL-15) are associated with α7-nAChR AAbs OD and that BD and SZ but not HC are associated to an increase in α7-nAChR AAbs OD (Supplementary Table 1).

### Unsupervised inflammatory marker-based clustering

**Fig. 2:**
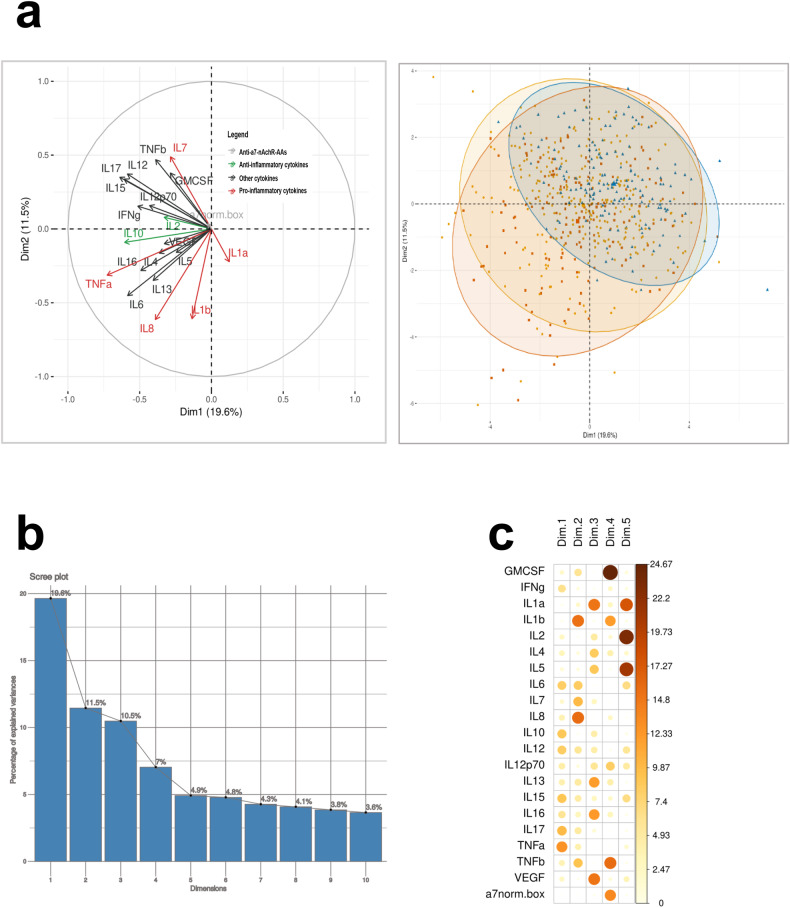
Principal component analyses (PCA) on serum immune-inflammatory markers. **a** Left panel: correlation circle including all the serum cytokines and anti-α7-nAChR AAbs; right panel: spatial distribution of diagnoses on the correlation circle (blue = HC; orange = BD; red = SZ). **b** Decomposition of explained variance by anti-a7-nAChR AAs (Table 2) principal components, the five first dimensions explained 53.5% of the variance. **c** Variables contribution for each dimension.

We then carried out further analysis using two-step unsupervised clustering. We first applied a Principal Component Analysis (PCA) to reduce the large number of variables into a small number of dimensions. The PCA revealed subjects with increased levels of pro-inflammatory cytokines, mostly patients, represented by the first dimension. Interestingly, no HC were present in this part of the plot, meaning that no HC have elevated concentrations of pro-inflammatory cytokines such as TNF-α (Fig. 2a, left and right panels). These findings confirmed that we cannot clearly distinguish SCZ and BD patients on their inflammatory profile whereas we can distinguish psychiatric patients from HC. To conserve a complete model for the two-step clustering, we have kept the first five dimensions which summed up to 53.5% of the explained variance in the model (Fig. 2b). The weight of every variable for each dimension is highlighted in Fig. 2c. Here the contribution of α7-nAChR AAbs was strong in the fourth dimension along with GM-CSF, IL-1β, and TNF-β.

### Inflammation-based two-step clustering: clinical scores, cytokine profiles, and serum antibodies

**Fig. 3:**
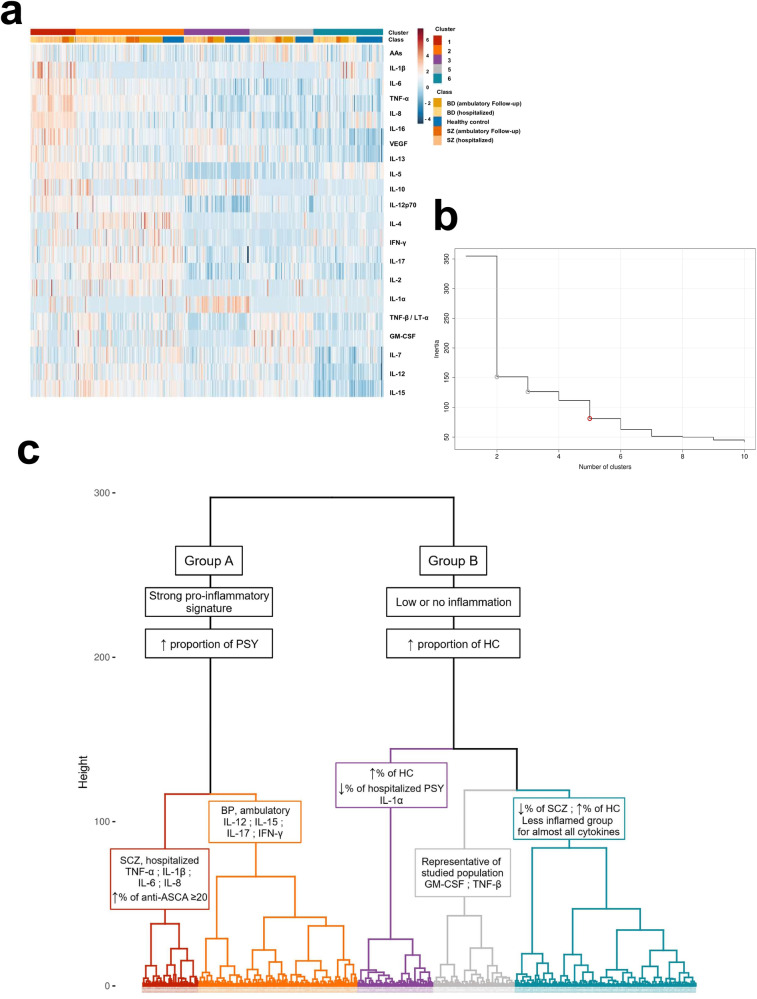
Hierarchical clustering defines 5 clusters based on immune-inflammatory profile. **a** Heatmap representation of inflammatory profiles within all the population (diagnoses are highlighted as follows: blue = HC, dark orange = hospitalized BD, light orange = ambulatory BD, dark red = hospitalized SZ, light red = ambulatory SZ). **b** Selection of the optimal number of clusters according to inertia. **c** Dendrogram illustrating cluster slicing and composition.

This unsupervised two-step clustering method allowed us to partition the subjects solely on the basis of their inflammatory profile and the OD of α7-nAChR AAbs without giving any clinical or sociodemographic information. First, the results confirm the data in the literature concerning the link between inflammatory status such as an increase in the pro-inflammatory cytokines TNF- α, IL-1ß, IL-6, and IL-8 and the occurrence of psychiatric pathology such as SCZ and BD. Indeed, the first step consisting in an unsupervised hierarchical ascendant classification approach first separated the sample into two majour groups: the group A with subjects with high levels of pro-inflammatory cytokines, and the group B with subjects with low concentrations of pro-inflammatory cytokines. The group A was composed of 85.8% of patients and 14.2% of HC, whereas the group B was composed of 65.8% patients and 34.2% HC (Fig. 3c). The second step allowed us to subdivide these groups into five clusters according to the optimum inertia and the visualization of the heatmap (Fig. 3a, b). Once the five clusters were identified, we studied their sociodemographic, clinical and infectious characteristics by comparing their proportion for discrete variables such as gender, tobacco status, the diagnoses, the positivity to previous infections, and the mean for continuous variables such as age and the scores on clinical scales (Table 2).

**Table 2:** Clinical and serological characteristics within clusters.

In the first line, the colors are the color code of clusters on the graphs. In the table, the color goes from far red to deep blue according to the standardized residual as demonstrated in the scale on the bottom of the table (far red = −3 ; deep blue = 3).

^a^Chisq-test for qualitative variables (color related to the standardized residuals).

^b^Kruskal–Wallis followed by Dunn’s test for multiple comparisons for quantitative variables (*P* values adjusted with Bonferroni method).

^#^Two-way ANOVA (status and clusters).

**Fig. 4:**
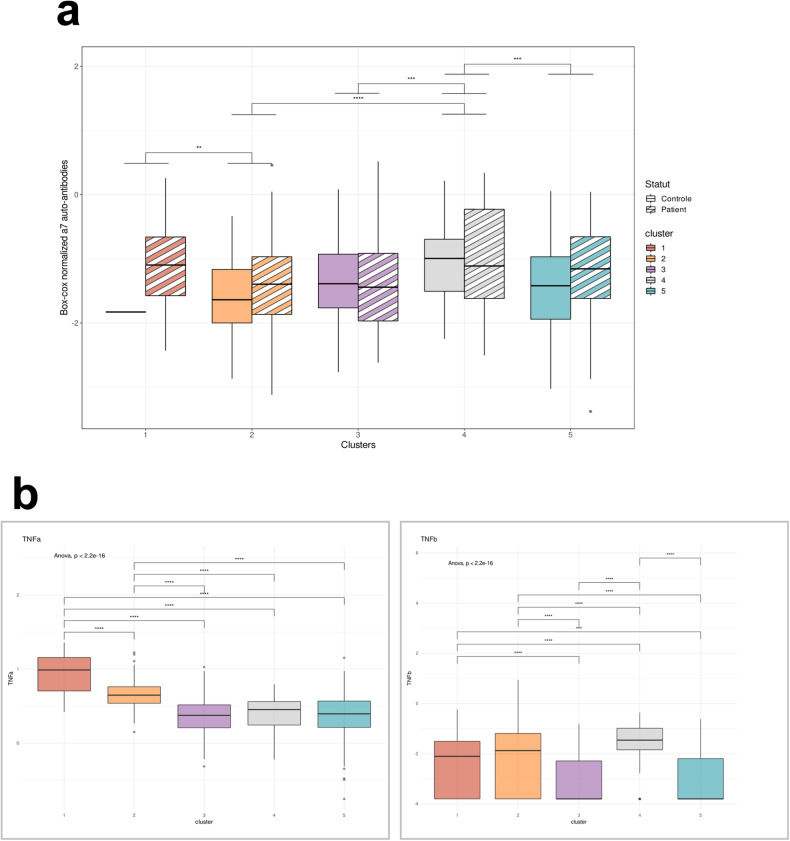
Anti-α7-nAChR AAbs and tumor necrosis factor in clusters. **a** Boxplot of anti-α7-nAChR AAb OD between clusters in healthy controls (striped bar) and psychiatric patients. **b** Levels of TNF-α (left panel) and TNF-β (right panel).

Interestingly, this 5-cluster partition has split the group A into two clusters (CL1=Red; CL2=Orange). These two clusters differ in terms of clinical features and inflammatory signatures. The CL1 is composed of 99% of psychiatric patients and has the highest percentage of acute patients (72%), and especially SZ (33.3%), compared to the CL2 that presents the highest proportion of ambulatory patients (53.8%), especially with BD (24.6%). Both clusters presented the lowest proportion of HC, which was congruent with the previous PCA. These two clusters also differ in inflammatory profile since the CL1 was marked by innate inflammatory cytokines, including TNF-α, IL-1β, IL-6, IL-8, IL-10, IL-13, and IL-16, classically found in acute inflammation, whereas CL2 showed increased levels of IFN-γ, IL12/23p40 and p70 as well as IL-15 that play a role in adaptative immunity. The group B was split into three groups with less pronounced inflammatory profiles. CL3 (purple) and CL5 (blue) have the highest proportion of HC (respectively, 37% and 37.1%), CL3 presented the lowest proportion of hospitalized patients and the lowest proportion of BD, and CL5 has the lowest proportion of hospitalized SCZ (7.8%) (Table 2). The CL3 was marked by increased levels of IL-1α, and CL5 was the cluster with the lowest levels of pro-inflammatory cytokines. Surprisingly, CL4 did not significantly differ from the whole study sample in terms of composition. However, the CL4 showed increased levels of the inflammatory cytokines GM-CSF and TNF-β-/LTα. According to our results, we did not find significant differences concerning psychotic and mood symptoms. However, the CL3 presented the highest score of GAF Symptoms (50.1 ± 20.0). Significant group effects were also found concerning the FAST and the GAF Handicap scores, however, the differences were not significantly different after *p*-value adjustments. CL1 and CL4 were the two clusters with the highest OD of anti-α7-nAChR AAbs (Table 2). Interestingly, they were both associated with an increase of the two TNF, the CL1 presented increased levels of TNF-α and CL4 increased levels of TNF-β (Fig. 4b). However, only CL4 presented the highest proportion of subjects significantly above the cutoff previously described (21.8%). In addition, the CL2 and the CL5 both presented a lower proportion of subjects above the cutoff despite completely different cytokine profiles and composition (Table 2). Finally, CL1 presented the highest proportion of subjects with serum antibodies against HSV-1 (73%), toxoplasma gondii (77%) and anti-saccharomyces (28%) compared to the four other clusters (Table 2). We decided to carry out a more detailed analysis in each cluster and compare the levels of anti α7-α7-nAChR AAbs between patients and HC inside each cluster. We found that the psychiatric patients and HC have similar levels within each cluster (Fig. 4a).

### Functional effects of patients’ serum on TNF-α release by human macrophages

**Fig. 5:**
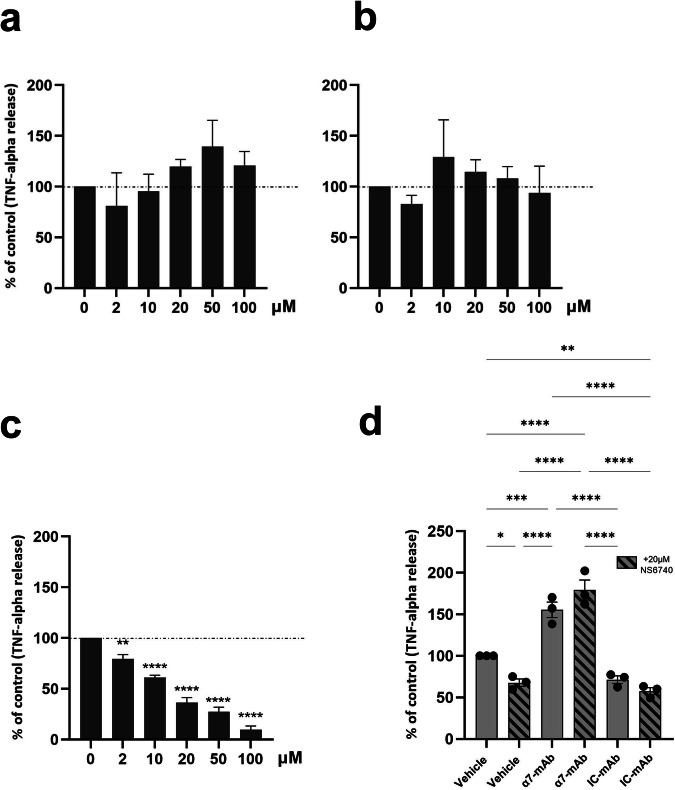
TNF-α release assay upon LPS challenge in macrophages incubated with nAChR drugs or Ab. **a** No effect of nAChR agonist PNU-282987 (*F* = 1173, *P* = 0,3774)* and **b** nAChR PAM (*F* = 0,6635, *P* = 0,6581)* compared to control. **c** Anti-inflammatory effect of the nAChR partial agonist NS6740 (*F* = 84,48, *P* < 0,0001)* after 6 h of stimulation with LPS *E. coli*. **d** Anti-α7-nAChR-mAb increases the LPS-induced TNF-α release in human macrophages compared to isotype control (IC-mAb, 53.80; *P* < 0,0001)*. *One-way ANOVA followed by Holm–Sidak’s post hoc test for multiple comparisons. Dashed line= release of TNF-α after 6 h of LPS challenge without any pharmacological agents (100%). For each condition, *N* = 3 independent experiments.

We then pursued our working hypothesis that increased levels of AAbs could increase the production of pro-inflammatory cytokines by blocking the α7 receptor on macrophages. As a proof-of-concept, we used the commercial mouse anti-human α7-nAChR mAb (MA53169) to study whether anti- α7-nAChR Abs could prevent the anti-inflammatory effect of a drug targeting the α7-nAChR. To decipher which drug was the most relevant for our study, we tested three compounds often used to study signaling or neuroinflammation mediated by the α7-nAChR: PNU-282987, a full agonist of the receptor, PNU-120596 a positive allosteric modulator (PAM) and NS6740, a partial agonist also known as “silent agonist” that maintains the α7-nAChR in a desensitized state [38, 39]. Our results showed that neither the full agonist nor the PAM was able to decrease TNF-α release by THP-1 macrophages after 6 h of stimulation with LPS (Fig. 5a, b). However, the silent agonist NS6740 decreased the TNF-α release in a concentration-dependant manner from 2 to 100 µM (Fig. 5c). For the next experiments, we decided to use the NS6740 at a concentration of 20 µM to evaluate whether an anti-α7-nAChR mAb could block its anti-inflammatory effect compared to its control isotype (IC-mAb), which is a non-specific mouse IgG1.

Our results show that incubation with anti-α7-nAChR mAb alone substantially increases the release of TNF-α by macrophages challenged with LPS for 6 h (+55%, *P* < 0.001, Fig. 1d), whereas the incubation with the IC-mAb did not significantly modify the release of TNF-α (−29%, *P* = 0.06). However, the incubation with the silent agonist NS6740 decreased TNF-α release in the control condition (−32%, *P* = 0.037) and the IC-mAb condition (−42%, *P* = 0.0066). However, the anti-inflammatory effect of NS6740 was abolished in cells pre-incubated with the specific anti-α7-nAChR mAb (+79%, *P* < 0.0001,) compared to the control (Fig. 5d).

## Discussion

Here, we have explored the link between AAbs against nAChRs, inflammation, and psychiatric disorders. These findings are based on the use of a novel ELISA system using a preparation of the pentameric extracellular domain of the human α7, including the main immunogenic region that represents a more sensitive alternative to the standard CBA carried out often on fixed cells that can give contradictory results [40]. Indeed, previous studies showed that paraformaldehyde can prevent the binding of toxin specific for nAChRs [41]. This could explain the controversial data on anti-nAChR AAbs. There are only two previous papers carrying out a related analysis. The study by Chandley et al. [32] used a similar approach to ours, employing a full-length α7 protein synthesized in wheat germ. Chandley et al. identified 23% of patients with such AAbs [32]. This is to be contrasted with the study of Hoffmann et al. [42]. Only one psychotic patient out of 711 subjects was positive for anti- α7-nAChR AAbs according to radioimmunoassay (RIA) with iodine 125-labeled α-bungarotoxin on membrane extract from transfected HEK293 cells expressing human α7-nAChR. Although using ELISA for the quantification of the monoclonal control antibody, they did not pursue this technique further. They also used a cell-based assay (CBA) on fixed and non-fixed HEK cells expressing the α7-subunit. The authors discuss the issue of having failed to detect more signal and consider that their use of α-bungarotoxin in the RIA competition might compete with the AAbs on binding to the α7 AChR.

Overall, our results provide additional evidence that immune-inflammatory dysregulation occurs in psychiatric patients and implicates AAbs targeting nAChRs. Here, we confirmed that such AAbs are in human serum and are increased in two groups presenting innate immunity signatures including the two forms of TNF-α and β that have been associated to autoimmune, neuropsychiatric and demyelinating diseases [8, 43–45]. These two cytokines are 30% homologous and members of the TNF superfamily genes located on chromosome 6p21.3 [46]. TNF-α is mainly produced by macrophages and is regulated by several subcellular pathways involving α7-nAChR in an auto- and paracrine fashion [47, 48]. Indeed, in wild-type mice, it has been shown that vagus nerve stimulation decreases the release of LPS-induced TNF-α release and that this effect is abolished in α7 knock-out mice [49]. Some studies suggest that α7-nAChR modulates TLR4 expression and thus regulates the response to LPS. So far, the role of nAChR in the regulation of TNF-β by lymphocytes remains unknown. TNF-β (also called LT-α) is a cytotoxic factor produced by activated lymphocytes that enhances their switch into NK cells [46, 50]. Its suppression by antibodies or genetical tools prevents acute and chronic experimental autoimmune encephalomyelitis and the induction of myasthenia gravis in mice [45, 50]. Concerning psychosis, TNF-α levels and TNF-β polymorphisms have also been documented [51] and constitute the low-grade inflammation observed in SZ, BD, and major depressive disorders [3].

Here, we found a significant anti-inflammatory effect only for the drug NS6740 and not for the positive allosteric modulator PNU-120596 in THP-1 macrophages. These results are in line with the fact that NS6740 but not PNU-120596 reduces TNF-α release in cultures of rat microglia challenged with LPS [52]. Besides, we did not find any anti-inflammatory effect of the full agonist PNU-282987, whereas others have reported the opposite [53, 54]. Both PNU molecules have been widely used for electrophysiology with cells expressing the neuronal α7-nAChR or directly on neurons. They are highly efficient to induce (PNU-282987) and maintain (combination PNU-282987/120596) nAChR currents [55]. However, some studies suggest that non-neuronal nAChR expressed on immune cells undergo different post-translational modifications compared to the neuronal receptor and do not exert the same ion channel role. The anti-inflammatory effect is differentially modulated between immune cell subtypes and species and might involve a metabotropic activity [56, 57]. The intracellular domain (ICD) of α7-nAChR contains sites for tyrosine phosphorylation and possibly interacts with G proteins. Studies using mutated α7-nAChR have shown that the mutations of tyrosine residues on the ICD have the same effect as kinase inhibitors and prevent the PNU-induced phosphorylation of ERK1/2, a pathway involved in the transcription of pro-inflammatory cytokines [58]. Growing evidence suggests that metabotropic activity occurs when the receptor is in a desensitized state which might explain the effectiveness of NS6740 [59]. Another main factor is the existence of a translational gap between humans and mice concerning α7-nAChR, the dupα7 [30]. This protein, specific to humans, is encoded by the *CHRFAM7A* gene and lacks a part of the extracellular domain. Dupα7 assembles with the α7-subunit and acts as a negative regulator of the α7-nAChR [60, 61]. Although the structure has not been characterized yet, mRNA studies have shown that its brain expression is altered in patients with psychosis [30], upregulated in epithelial cells and downregulated in THP-1 monocytes under LPS stimulation [62, 63]. As the NS6740 maintains the nAChR in a long-lasting desensitized state, we can hypothesize that this conformation activates JAK2/STAT3 pathways [64, 65], and that this effect is blocked by a specific anti-α7 mAb that might provoke steric hindrance, and then prevents the access to the binding sites of the receptor or modify the receptor conformation that could impede the intracellular cascade. These preliminary results suggest that α7-nAChR targeting Ab could have a deleterious role in the regulation of inflammation and could maintain a pro-inflammatory state. The next step to reinforce these results will be the use of purified IgG or recombinant antibodies obtained from patient blood samples, since in our model we use monoclonal IgG specifically targeting the extracellular part of the receptor. Beside, our results also reinforce the infectious hypothesis of psychotic disorders [66, 67]. We can suppose that repeated infections promote the circulation of damaged tissue and cell debris and foster antigen-presenting cells to present parts of nAChR to lymphocytes and thus lead to AAb production as shown in other diseases [68]. Here, by using a clustering algorithm, we have isolated one cluster only composed of psychiatric patients with severe symptoms, infectious stigmas, pro-inflammatory biosignatures and high anti-α7-nAChR AAbs.

We assume that circulating AAbs targeting nAChR and thus a low-grade inflammation could alter the protective capacity of the blood-brain barrier or the intestinal mucosa and thus promote the passage of antibodies and cytokines into the CNS. In this context, further investigation involving the measure of IgM subtypes have to be considered. Indeed, IgM subtypes are produced in the first days following an infection and are therefore indicative of a recent contact with a pathogen. This information would complement the data given by the cytokine measurements, which could be elevated due to an ongoing infection.

These mechanisms might be underlined by genetic predispositions involving, for example, the HLA system [69, 70], and require further explorations including the study of genetics and brain inflammation, which is very limited in the present cohort. Indeed, it would be crucial in the next studies to include a disease-control population without psychiatric condition in our clustering model which is here one of the main limitations.

Furthermore, future studies using cloned antibodies from psychiatric patients are needed. The use of this technology has enabled major advances to be made in understanding the physio-pathogenic mechanisms underlying AAbs from patients suffering from inflammatory diseases of the CNS, such as autoimmune encephalitis [71]. Using this technique to characterize IgG specifically directed against nAChRs would enable us to study in greater depth the epitope targeted by the antibodies and also to determine whether they would be capable of inducing the same effects as the commercial mAb used in this study. It would be very interesting to compare recombinant mAbs from patients and controls from the five clusters to establish whether only antibodies from CL1 patients would be amplifiers of inflammation.

To conclude, our findings have implications for immuno-psychiatry and provide new evidence that the cholinergic pathway and the allosteric properties of nAChRs represent a promising target in drug development for SCZ, BD, and beyond.

### Supplementary information

Supplement

## Data Availability

The biological data, protocols, and code used in the present study are available upon reasonable request.
