# Supplementary material for: Serum autoantibodies against α7-nicotinic receptors in subgroups of patients with bipolar disorder or schizophrenia: clinical features and link with peripheral inflammation
Source: Transl Psychiatry. 2024 Mar 14;14:146. doi: 10.1038/s41398-024-02853-8 (PMC10940727; doi:10.1038/s41398-024-02853-8)
Supplement: Supplementary file 1 — Supplement [file 41398_2024_2853_MOESM1_ESM.pdf]

Supplementary Figures & Tables

Supplementary Table 1:

Multiple linear regression: Association between anti-α7-nAChR-AAb rates, cytokines, age, sex, and diagnosis in the whole population

	OUTCOME		
**PREDICTORS**	Estimates	CI	p
**(INTERCEPT)**	-0.49	-0.57 – -0.41	<0.001
**GMCSF**	-0.06	-0.15 – 0.03	0.170
**IFNG**	0.00	-0.00 – 0.00	0.289
**IL1A**	-0.00	-0.00 – 0.00	0.710
**IL1B**	0.04	-0.02 – 0.10	0.149
**IL2**	**0.05**	**-0.00 – 0.11**	**0.070**
**IL4**	**-0.62**	**-1.17 – -0.06**	**0.031**
**IL5**	**0.02**	**-0.00 – 0.03**	**0.076**
**IL6BIS**	0.01	-0.01 – 0.02	0.367
**IL7**	**0.00**	**0.00 – 0.01**	**0.028**
**IL8**	0.00	-0.00 – 0.00	0.955
**IL10**	-0.01	-0.02 – 0.01	0.485
**IL12**	0.00	-0.00 – 0.00	0.165
**IL12P70**	**-0.03**	**-0.07 – 0.00**	**0.069**
**IL13**	0.00	-0.01 – 0.02	0.568
**IL15**	**-0.04**	**-0.07 – -0.01**	**0.005**
**IL16**	-0.00	-0.00 – 0.00	0.161
**IL17**	-0.00	-0.00 – 0.00	0.987
**TNF-A**	-0.00	-0.01 – 0.00	0.155
**TNF-B**	0.06	-0.04 – 0.16	0.206
**VEGF**	0.00	-0.00 – 0.00	0.177
**AGE**	0.00	-0.00 – 0.00	0.769
**SEXE [M]**	0.02	-0.02 – 0.05	0.313
**DIAGNOSTIC [BP]**	**0.08**	**0.03 – 0.12**	**0.001**
**DIAGNOSTIC [SZ]**	**0.07**	**0.02 – 0.12**	**0.011**
**OBSERVATIONS**	584		
**R^2 / R^2 ADJUSTED**	0.081 / 0.042		

Supplementary Figure 1: Data normalization after Box-Cox transformation: Left panels are raw data and right panels are transformed data. (a) Anti-α7-nAChR-AAbs (b) Cytokines (c) PCA plots with AAbs and cytokines

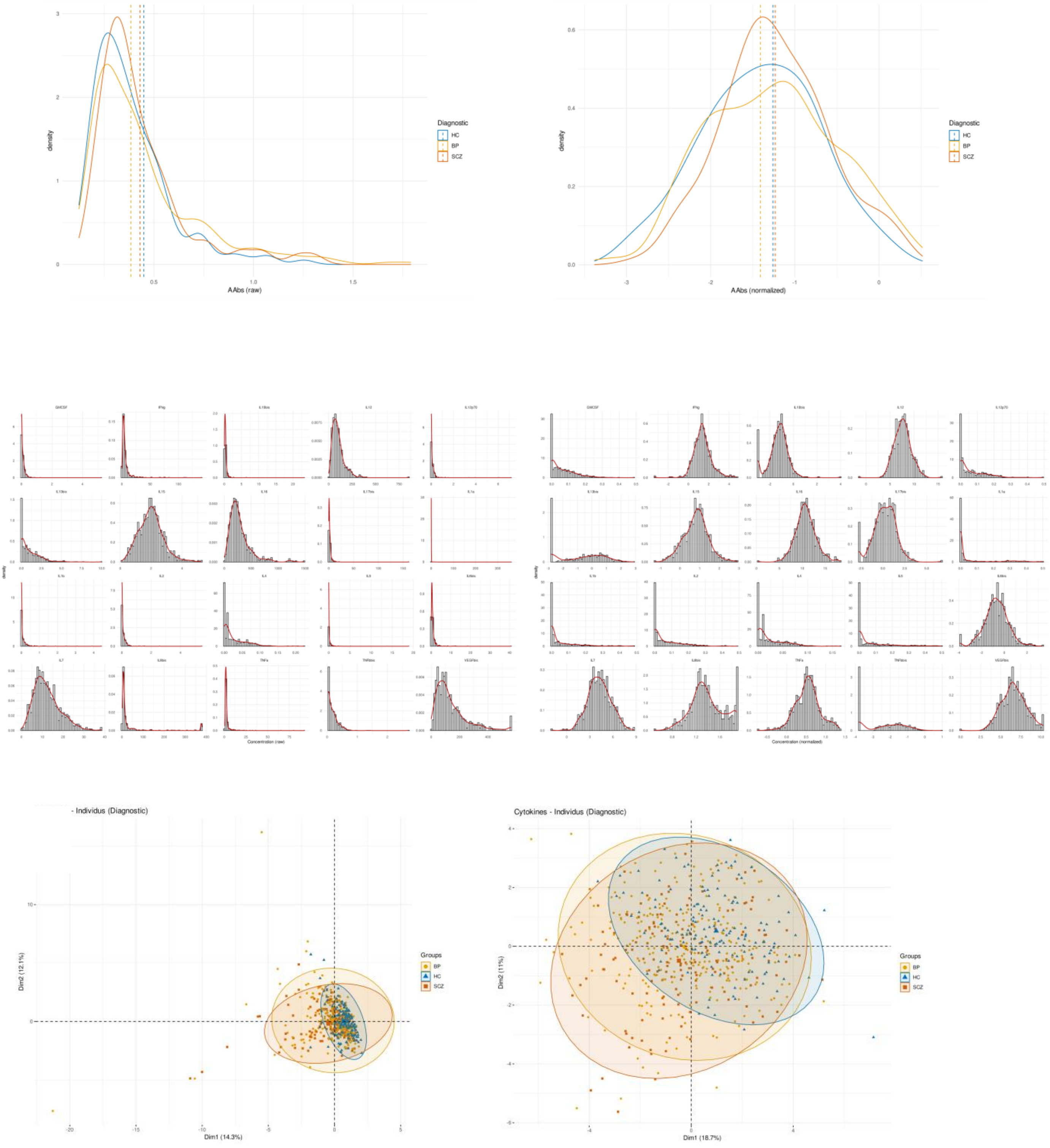

Supplementary Figure 2: Schematic representation of lentiviral vectors used to create CHO-K1 cells expressing the human a7 nAChR :The cDNA of the human a7 nAChR and the two chaperone proteins Nacho and Ric3 was cloned under the control of the CMV promoter and then used to infect cells with a ratio of 5:1:1. Cells were sorted by FACS, individual cells were cloned and clones expressing high levels of a7 nAChR at the plasma membrane were selected by bungarotoxin immuno-staining.

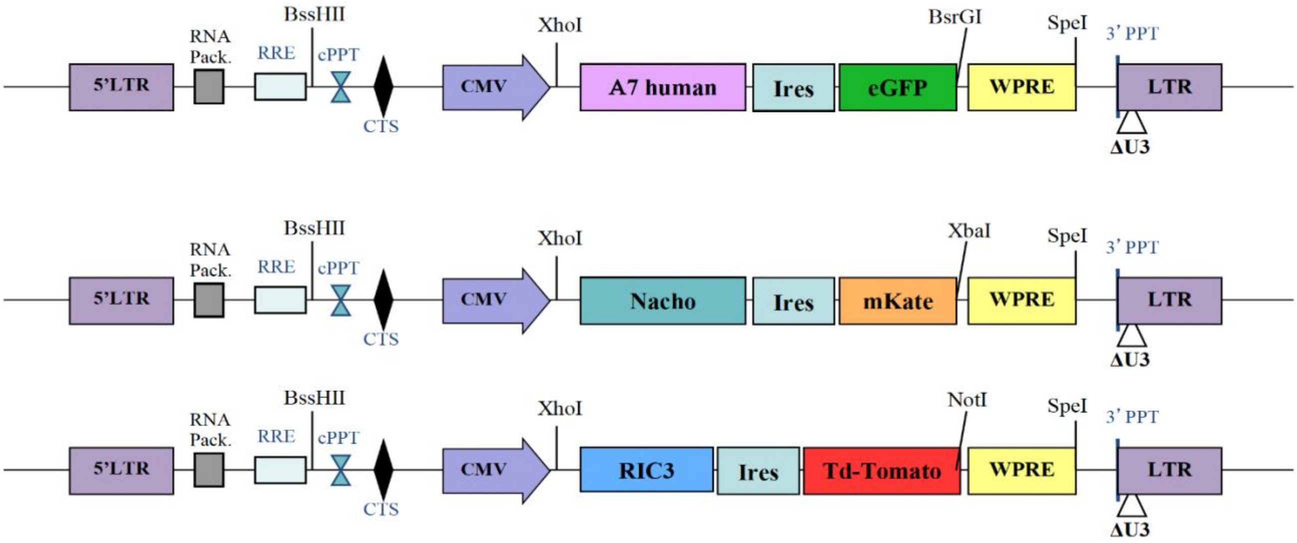

Supplementary materials

Human α7-nAChR expression in CHO

The plasmid constructs were used to prepare lentiviral particles as already described in Maskos and al. (*Nature* **436**, 103–107 (2005)).

The viral particles are directly added to the cells during their seeding at 10^5 cells/well (DMEM/F12, Gibco, Grand Island, NY) and respecting a ratio of 5 particles of a7 nAChR for 1 particle of each chaperone protein (10µg of p24 for a7 nAChR particles and 2µg for chaperones).

The day after fresh medium was added to the cells. After 3 days of expression, cells were sorted by FACS (FACS-Aria III; BD Life Sciences, San Jose, US) and cells expressing the 3 reporter genes were seeded in 96 well plates to obtain individual clones. Clones expressing high levels of a7 nAChR at the plasma membrane were selected by bungarotoxin immuno-staining. Briefly, aliquots of clones were fixed with 2% PFA (Santa Cruz Biotechnology, Inc ; Dallas, Texas) for 10 min then rinsed with PBS 1x, saturated 1h at room temperature with PBS 1x + 5% normal horse serum (Gibco, Grand Island, NY) + 1% fish skin gelatin (Sigma, Saint Louis, MO, US). Fluorescent bungarotoxin conjugated (Invitrogen™, Massachusetts, US) was finally incubated 3h at room temperature in PBS 1x + 1% normal horse serum (Gibco, Grand Island, NY) + 0.2% fish skin gelatin (Sigma, Saint Louis, MO, US).
